# Toward On‐Chip Nascent All‐van‐der‐Waals Polarization Optical Elements for Nanoscale Photonic Applications

**DOI:** 10.1002/nap2.70327

**Published:** 2026-10-24

**Authors:** Dmitrii Litvinov, Ilia Begichev, Christoph Reuter, Changyi Chen, Iakov Reznikov, Maksim Sargsyan, Sergey Y. Grebenchuk, Zdeněk Sofer, Makars Šiškins, Kostya S. Novoselov, Alexey I. Berdyugin, Maciej Koperski, Davit A. Ghazaryan

**Affiliations:** ^1^ Institute for Functional Intelligent Materials National University of Singapore Singapore; ^2^ Department of Materials Science and Engineering National University of Singapore Singapore; ^3^ Laboratory of Advanced Functional Materials Yerevan State University Yerevan Armenia; ^4^ Department of Inorganic Chemistry University of Chemistry and Technology Prague Prague Czech Republic; ^5^ School of Physics and Astronomy University of Southampton Southampton UK; ^6^ Department of Physics National University of Singapore Singapore

**Keywords:** all‐vdW polarization optical components, birefringent polarization‐conversion elements, in‐plane anisotropy, low‐symmetry vdW crystals, on‐chip nanophotonics

## Abstract

The integration of polarization‐control elements into nanoscale photonic circuits remains a central challenge for modern on‐chip optical technologies, which call for continuous miniaturization. Van der Waals (vdW) crystals provide a versatile platform for their creation because of strong optical anisotropy, low loss accompanied by high refractive indices, and atomically precise heterostructure assembly capabilities. In this work, we present an approach towards the creation of all‐vdW on‐chip birefringent polarization‐conversion elements operating in the near‐infrared (NIR) spectral region, realized by uniquely twisted $ReSe_{2}/\alpha -MoO_{3}$ heterostructures placed on a $Si/SiO_{2}$ platform. Our proof‐of‐principle stacks demonstrate up to 64.5 % average degree of circular polarization (DoCP) quantitatively reproduced by finite‐difference time‐domain (FDTD) simulations. The latter reveals critical roles of layer thickness, twist angle, and Fabry–Perot interference effects in the determination of polarization conversion efficiency, which yields DoCP values close to unity for a layer thickness of about twice that of zero‐order quarter‐wave plate conditions of a free‐standing slabs. Our findings establish a practical route toward fully integrated all‐vdW polarization optical elements, providing a foundation for nanoscale photonic architectures entirely based on layered materials.

## Graphical abstract

Twisted ReSe_2_/*α*−MoO_3_ van der Waals heterostructures pave the way towards next‐generation polarization nanophotonic applications, where combining two biaxial layers—a triclinic source of linearly polarized emission and an orthorhombic polarization‐conversion slab—establishes a practical route for fully integrated on‐chip all‐van der Waals polarization elements with high degrees of circular polarization.

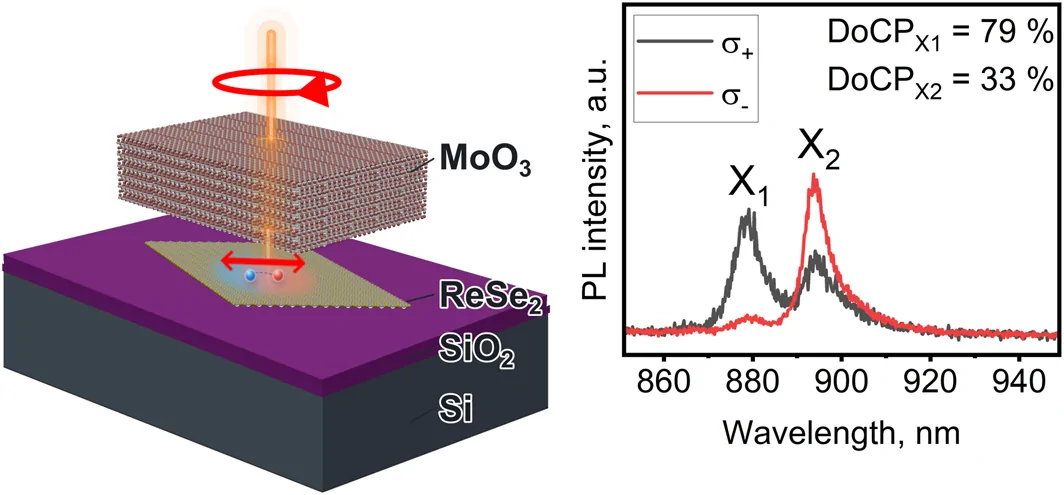

## 1 Introduction

Since the isolation of graphene [1], the developments in the field of two‐dimensional (2D) crystals paved the way for the design and control of their electronic, optoelectronic, and magnetic properties through atomically precise fine‐tuning of twist angle, 2D layer thickness, composition, and arrangement within vertically stacked few‐layer vdW heterostructures [2, 3, 4, 5, 6, 7].

Transferring technological recipes applied to graphene toward optically thick counterparts of 2D crystals established a powerful platform for the advancement of visible‐to‐infrared (Vis‐*to*‐IR) spectral region nanophotonic and integrated optics applications, mostly because of the emerging strong light–matter interaction effects and highly tunable optical responses that are accompanied by high refractive indices [8, 9, 10, 11, 12, 13, 14, 15]. More recently, the latter research scope extended to include biaxial vdW crystals, further launching strong polarization‐based phenomena [16, 17, 18]. These exhibit in‐plane optical anisotropy, which arises along with an out‐of‐plane one (based on a shear layered structure) from the reduced crystal symmetry, providing an additional degree of freedom to control and tune the fundamental properties of electromagnetic waves interacting with matter, such as polarization, propagation direction, and phase. This makes them an attractive platform for the realization of ultrathin Vis‐*to*‐IR polarization optical elements, such as linear polarizers, subwavelength half‐ and quarter‐wave plates [18, 19, 20, 21], and others.

Black phosphorus is a vivid example of a biaxial vdW crystal, which exhibits strong in‐plane optical anisotropy within nanometer‐scale thicknesses due to its direction‐dependent dielectric response [22]. Its puckered crystal structure demonstrates an emergence of pronounced linear dichroism between armchair and zigzag axes, providing control of the polarization state [23]. However, it is not optically transparent even in the few‐layer limit, thus, the strong in‐plane optical anisotropy uncovered in low‐symmetry transition‐metal or metalloid chalcogenides [18, 24, 25, 26, 27, 28, 29], such as GeS, $GeS_{2}$, $TiS_{3}$, and $As_{2}S_{3}$, made another important leap on the path toward ultrathin polarization optical elements owing to their transparency in the Vis, NIR and mid‐IR spectral regions. Other examples of vdW crystals exhibiting strong in‐plane optical anisotropy that have attracted significant attention include wide‐bandgap semiconductors [30, 31], such as $\alpha -MoO_{3}$, and distorted lattice crystals [32, 33, 34, 35, 36, 37], such as $ReS_{2}$ and $ReSe_{2}$, or specimens of the oxyhalide family, such as $MoOCl_{2}$ and $NbOCl_{2}$. Notably, the enormous in‐plane anisotropy in the latter, though typically accompanied by significant optical losses arising from directional Peierls distortion in the lattice, enables deep‐subwavelength ultraviolet‐*to*‐NIR polarization optical elements.

Remarkably, another extension of technological recipes applied to graphene toward vdW heterostructures composed of optically thick layers, where the same degrees of freedom (tunability of layer thickness, twist angle, composition, and arrangement) are intact, further allowed designing more sophisticated nanoscale polarization optical elements and nanophotonic components, such as beam splitters, chiral circular polarizers, and magic‐angle or moiré photonic devices [24, 32, 38, 39, 40, 41, 42, 43]. However, despite such rapid progress, recent works mainly focus on free‐space optical configurations; in practice, concrete nanophotonic applications require miniaturization and direct integration into on‐chip optical circuits.

In this work, we present a step‐by‐step approach toward the creation of fully integrated all‐vdW on‐chip birefringent polarization‐conversion elements operating in the NIR spectral region. Our results establish a straightforward recipe for the integration of uniquely twisted $ReSe_{2}/\alpha -MoO_{3}$ heterostructures with $Si/SiO_{2}$ chips, where in‐plane anisotropy of orthorhombic $\alpha -MoO_{3}$, which serves as a polarization‐conversion layer, enables the transition of linearly polarized excitonic emission from a triclinic $ReSe_{2}$ layer into a source of circularly polarized emission. Our proof‐of‐principle stacks show average DoCP up to 64.5 % for polarization‐conversion layers of 1.39 μm in thickness, when the quarter‐wave plate condition for free‐standing slabs is achieved at 2.85 μm. Moreover, our FDTD simulations quantitatively reproduce the experimental observations with good agreement, elucidating the critical influence of layer thickness, twist angle, and Fabry–Pérot interference on polarization conversion efficiency, where DoCP reaches values exceeding 99% for optimized parametrization requiring about half the thickness of that satisfying the zero‐order quarter‐wave plate condition in free‐standing slabs.

## 2 Results and Discussion

The schematics of the device structure composed of two anisotropic biaxial vdW layers are presented in Figure 1A. In a twisted vdW stack assembled on top of $Si/SiO_{2}$ chip, the bottom low‐symmetry layer acts as a source of linearly polarized light, whereas the top orthorhombic slab acts as a polarization‐conversion layer. For the emitting source, we chose $ReSe_{2}$ due to its triclinic (lowest‐symmetry) crystal structure (see Figure 1B, top), forcing excitonic optical dipoles [44, 45] to align along a specific in‐plane direction, resulting in nearly 100% degree of linear polarization (DoLP). An example of a low‐temperature photoluminescence (PL) spectrum (see Section 4) acquired from a bare 5.6‐nm‐thick $ReSe_{2}$ sample along with its polarization dependence is shown in Figure 1B, bottom. The plot reveals two spectral resonances at 879.4‐ and 894.8‐nm NIR wavelengths, denoted as $X_{1}$ and $X_{2}$, corresponding to A and B excitonic emissions with distinct polarization directions exhibiting $77^{o}$ rotation mismatch with respect to each other. Next, as a polarization‐conversion layer, we selected a multilayer of $\alpha -MoO_{3}$: an in‐plane anisotropic vdW crystal transparent in a broad Vis‐to‐IR spectral region, with an optical response governed by the pronounced structural anisotropy, that is, an orthorhombic crystal structure. Its schematics and optical dispersion showing distinct responses along all crystallographic axes for a multilayer sample are shown in Figure 1C. The optical constants, extracted from Mueller Matrix (MM) studies (see Section 4), reveal pronounced anisotropy throughout the measured spectral region along with substantial birefringence in both in‐plane and out‐of‐plane directions, in good agreement with previously reported values [46]. In particular, at NIR spectral region of interest (870–920 nm, corresponding to the excitonic emission wavelengths of $ReSe_{2}$), $\alpha -MoO_{3}$ exhibits an in‐plane anisotropy of $\Delta n_{xy}$ = $n_{x}-n_{y}\approx 0.08$, which was exploited as a cornerstone to convert linearly polarized excitonic emission from the bottom source layer into a circular polarized state.

**FIGURE 1:**
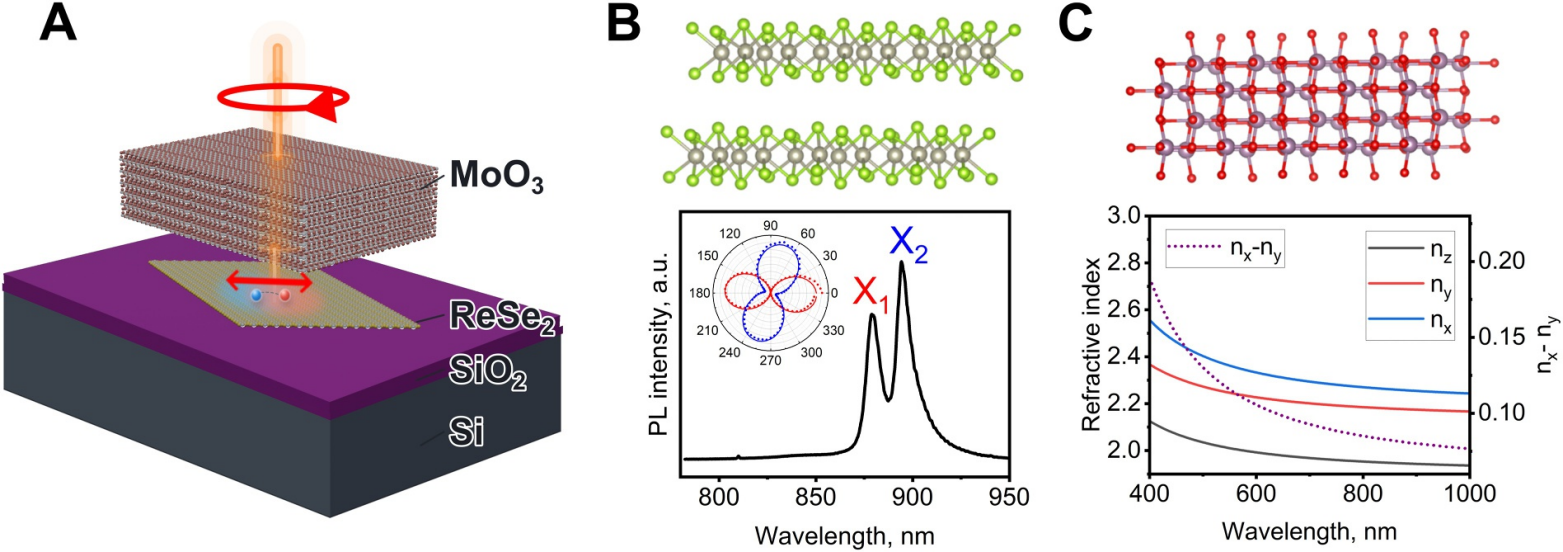
All‐vdW NIR spectral region on‐chip polarization‐conversion elements. (A) Schematic illustration showing the design of the device composed of prealigned $ReSe_{2}$/$\alpha -MoO_{3}$ vdW heterostructure positioned on top of $Si/SiO_{2}$ chip. (B) Schematic illustration of the triclinic crystal structure (top, side view) and low‐temperature PL spectrum (bottom) of $ReSe_{2}$. Inset displays the angular linear polarization dependence of A and B excitonic state emissions indicated as $X_{1}$ and $X_{2}$. (C) Schematic illustration of the orthorhombic crystal structure (top, top view) and measured optical constants (bottom) of $\alpha -MoO_{3}$ in the 400‐ to 1000‐nm spectral region.

The control of an alignment between emitted light's polarization and the slow axis of polarization‐conversion $\alpha -MoO_{3}$ layer is crucial for the achievement of a circularly polarized state and enablement of all‐vdW on‐chip polarization optical elements. Therefore, the slow axis of $\alpha -\mathrm{Mo}O_{3}$, that is, the corresponding in‐plane crystallographic direction, should be identified prior to the assembly of a vdW stack or controlled in situ in more sophisticated on‐site twistable architectures [47, 48]. Here, using polarization‐resolved Raman spectroscopy (see Section 4 and Figure 2A), similar to Ref. [49], using the strongest $A_{g}^{a}$ mode's polarization dependence, one may approximate the angular dependence of $\alpha -MoO_{3}$’s intensity in the copolarized geometry as $I_{\|}\left(\theta \right)∼\cos^{2}\;\theta$.

**FIGURE 2:**
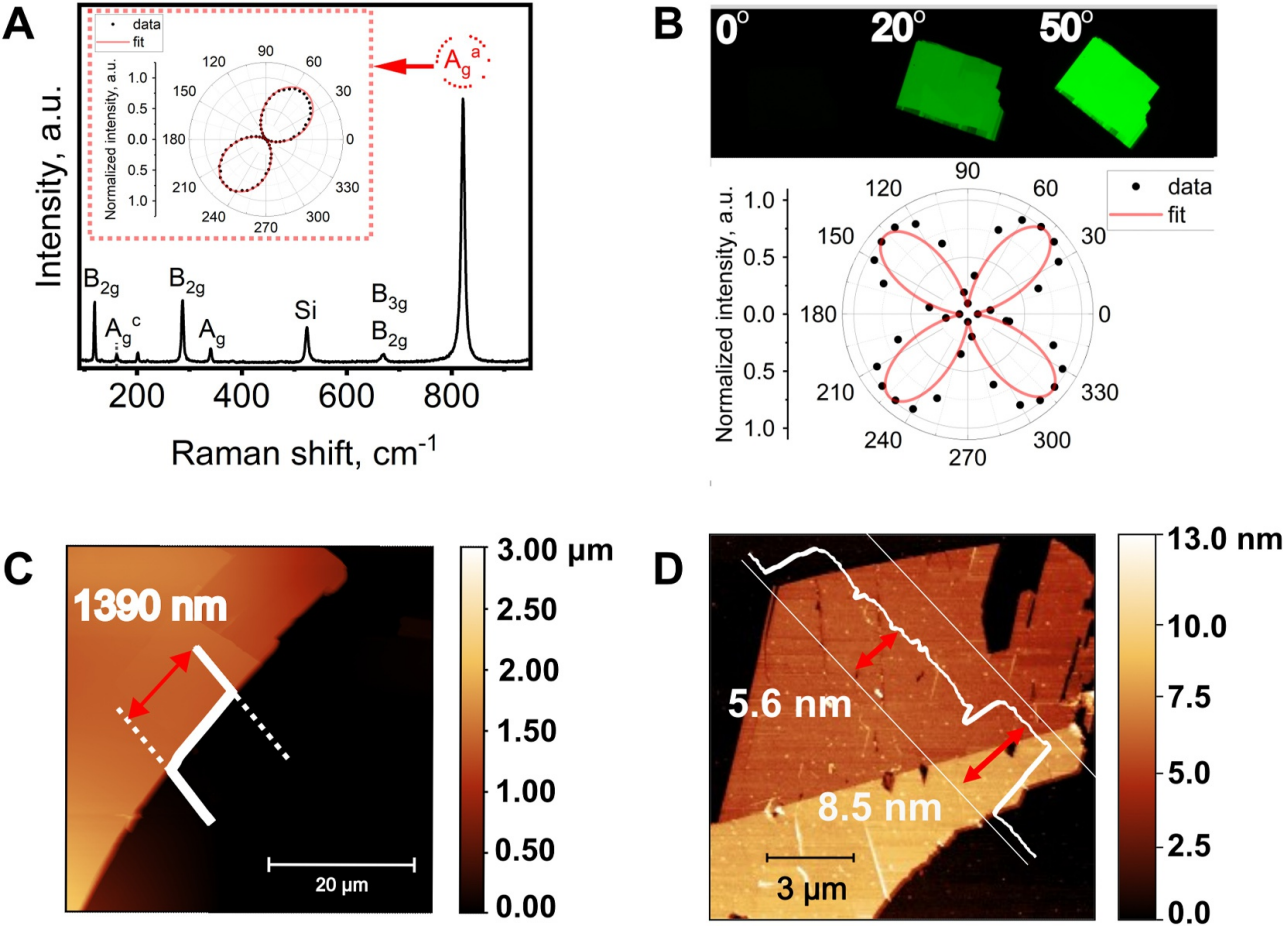
Identification of crystallographic axes of orthorhombic $\alpha -MoO_{3}$ and thickness characteristics of biaxial layers selected for the design of the on‐chip polarization conversion elements. (A) Unpolarized Raman spectra of a representative $\alpha -MoO_{3}$ multilayer illustrating $A_{g}$ and $B_{g}$ vibrational modes. The polar diagram in the inset shows the angular polarization dependence of the strongest $A_{g}^{a}$ mode obtained in copolarized geometry. (B) Polarization‐resolved Vis micro‐reflection response of $\alpha -MoO_{3}$. The top panel displays reflection micrographs at $0^{o}$, $20^{o}$ and $50^{o}$ rotations with respect to crystallographic axes. The bottom panel shows the full angular dependence of reflection intensity exhibiting a four‐lobe pattern. (C and D) AFM maps and thickness profiles (shown by red arrows) corresponding to cross‐sections of $\alpha -MoO_{3}$ (C) and $ReSe_{2}$ (D). All the samples presented throughout the figure were placed onto $Si/SiO_{2}$ substrates.

The latter exhibits a twofold symmetry that can be fitted by a simple relation of $\cos^{2}\;\theta$ with maxima oriented along the x‐axis as shown in the inset of Figure 2A. Another way to identify the crystallographic axes in $\alpha -MoO_{3}$ is through polarization‐resolved Vis micro‐reflection measurements (see Section 4). In the case of the cross‐polarized geometry, the intensity of the reflected light can be given as follows:

(1) $$I_{⊥}\left(\theta \right)\;\propto \;|A_{⊥}{|}^{2}=\frac{|r_{y}-r_{x}{|}^{2}}{4}\sin^{2}2\theta ,$$

where $r_{x}$ and $r_{y}$ are reflection coefficients of polarized light along x and y crystallographic axes. Here, because of an in‐plane optical anisotropy of $\alpha -MoO_{3}$ (Figure 1C, bottom), $r_{y}-r_{x}\;\neq \;0$; therefore, four lobes in the angular dependence are expected with the minima pointing along crystallographic axes (see Figure 2B). The top panel shows an example of the sample's reflection micrographs measured at $0^{o}$, $20^{o}$, and $50^{o}$ with respect to the axes directions, demonstrating nearly zero intensity in the case of the former while revealing a bright image in the case of the latter. The full angular dependence of the reflection intensity, along with its fitting for a representative $\alpha -MoO_{3}$ sample, is shown in the bottom panel of Figure 2B.

The following critically important parameter in the design of on‐chip all‐vdW polarization optical elements is the thickness of the polarization‐conversion layer, which governs the phase retardation between the two orthogonal directions. The quarter‐wave plate condition is fulfilled when $\frac{2\pi }{\lambda }\;\Delta n_{xy}\;d+\delta_{FP}=\pi /2$, where d is the thickness of an in‐plane anisotropic $\alpha -MoO_{3}$ layer, Δ $n_{xy}$ = $n_{x}-n_{y}$ is its in‐plane birefringence, and $\delta_{FP}$ is the thickness and proximity dependent Fabry–Pérot interference contribution to phase retardance. To estimate the latter, we first assume that light propagates freely through the polarization conversion layer and evaluate the phase retardance for the air/$\alpha -MoO_{3}$/air structure using the recent implementation of Berreman's anisotropic transfer matrix method (TMM) [50, 51]. Supporting Information S1: Figure S1 presents the corresponding results indicating two thicknesses of 2.85μm and 2.90μm achieving a full $90^{o}$ phase‐shift for a free‐standing $\alpha -MoO_{3}$‐based quarter‐wave plate. Next, we choose representative near‐micron‐thick $\alpha -MoO_{3}$ polarization‐conversion layer and $ReSe_{2}$ emissive layer for an assembly of our on‐chip all‐vdW polarization element and perform atomic‐force microscopy (AFM) scanning (see Section 4) to obtain the thicknesses of individual layers prior to stacking. Figure 2C,D (or Supporting Information S1: Figure S2) presents the corresponding results for Device 1 (or Device 2) with thicknesses of 1390 nm (or 910 nm) for $\alpha -MoO_{3}$ and 5.6 nm (or 70 nm) for $ReSe_{2}$ samples.

Figure 3A (or Supporting Information S1: Figure S3A) displays the optical micrograph of our proof‐of‐principle device, that is, twisted vdW heterostructure assembled (see Section 4) on top of $Si/SiO_{2}$ chip, for a case of preidentified direction of linear $A\;\left(X_{1}\right)$ excitonic state's polarization in the selected $ReSe_{2}$ layer (by measuring polarization‐resolved PL response, similar to data shown in inset of Figure 1B, bottom) and the slow axis of selected $\alpha -MoO_{3}$ multilayer (by measuring polarization‐resolved Raman response, similar to data shown in inset of Figure 2A), with a twist angle of $41^{o}$ (or $21^{o}$ for Device 2) between them. The polarization‐resolved PL mapping (see Section 4) of DoCP = $\left(I_{\sigma_{+}}-I_{\sigma_{-}}\right)/\left(I_{\sigma_{+}}+I_{\sigma_{-}}\right)$ of the resulting twisted vdW stack shows that a bare $ReSe_{2}$ demonstrates nearly zero values (Figure 3B or Supporting Information S1: Figure S3B for Device 2). An example of PL spectra for $\sigma_{+}$ and $\sigma_{-}$ circular polarization states acquired from this region is shown in Figure 3C (or Supporting Information S1: Figure S3C). On the other hand, in our twisted $ReSe_{2}$/$\alpha -MoO_{3}$ stack, we observe PL response of both excitonic emissions to be elliptically polarized. PL spectra from this region for $\sigma_{+}$ and $\sigma_{-}$ circular polarization states, with a maximum local value of 79% (see star‐marked position in Figure 3A), are presented in Figure 3D (see Supporting Information S1: Figure S3D for an example of PL spectra from Device 2). Note the stronger polarizability of $A\;\left(X_{1}\right)$ excitonic state emerging due to more preferable alignment condition of $41^{o}$ ($62^{o}$ for $B\;\left(X_{2}\right)$ state) with respect to the slow axis of $\alpha -MoO_{3}$. The resulting DoCP collected in the vicinity of both excitonic wavelengths from the twisted vdW heterostructure region shows average elliptical polarization of 64.5% (or 37%) and 36.5% (or 22.5%) for $A\;\left(X_{1}\right)$ and $B\;\left(X_{2}\right)$ excitonic state emissions (Figure 3E; see Supporting Information S1: Figure S3E for Device 2).

**FIGURE 3:**
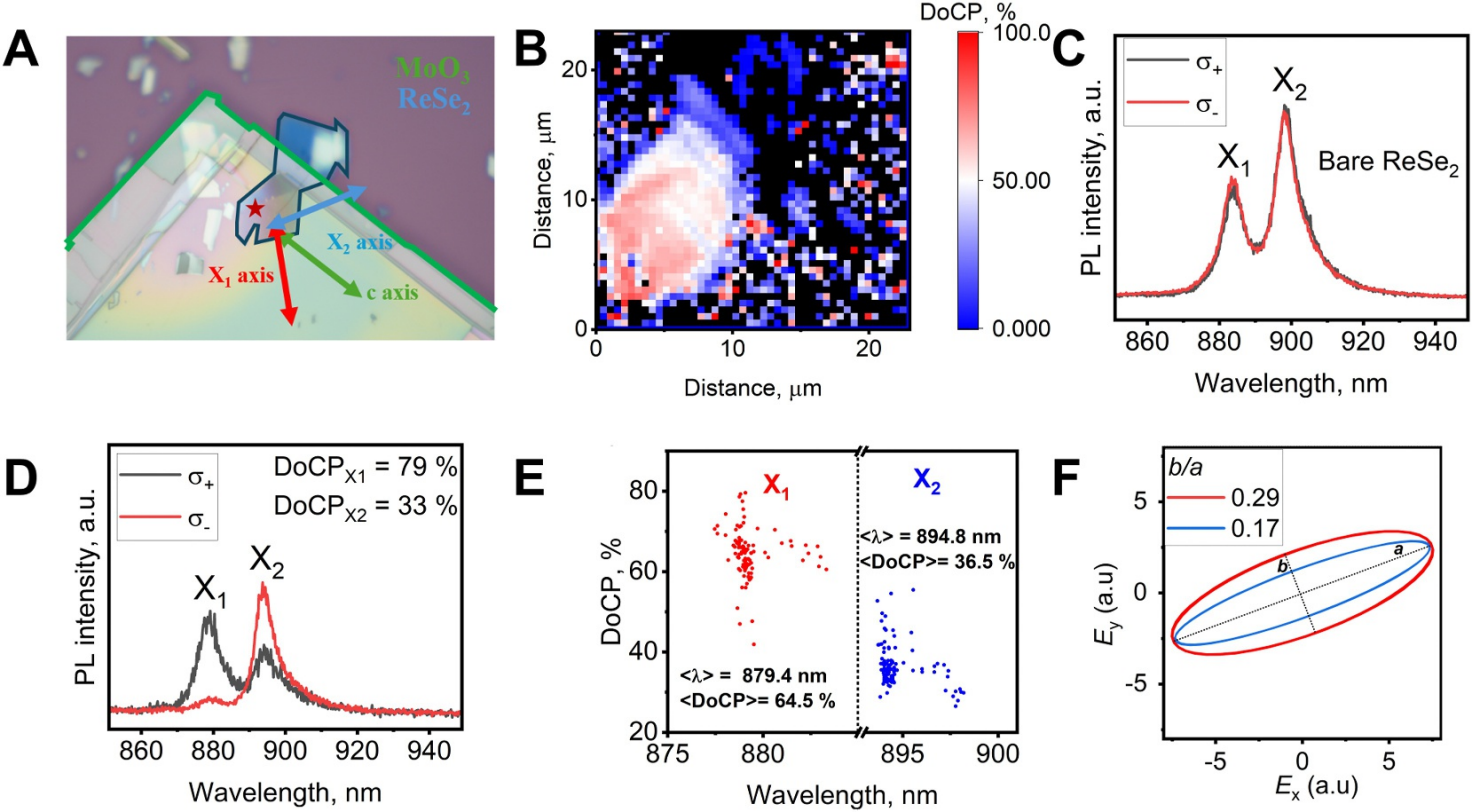
Probing DoCP in $ReSe_{2}$/$\alpha -MoO_{3}$ twisted heterostructure assembled on top of $Si/SiO_{2}$ chip. (A) 50 X optical micrograph of the proof‐of‐principle device, Device 1. The blue and red arrows denote the linear polarization direction of $A\;\left(X_{1}\right)\;\mathrm{and}\;B\left(X_{2}\right)$ excitonic emission from $ReSe_{2}$, whereas the green arrow shows the slow axis of $\alpha -MoO_{3}$ layer. (B) DoCP map collected all over the sample, including bare $ReSe_{2}$ and substrate. Circularly polarized $\sigma_{+}$ and $\sigma_{-}$ state PL spectra acquired from (C) bare $ReSe_{2}$ sample of 5.6 nm in thickness and (D) $ReSe_{2}$/$\alpha -MoO_{3}$ twisted heterostructure from a point marked by a star in (A). Excitonic states are denoted as $X_{1}$ and $X_{2}$. (E) DoCP histograms collected from the twisted stack as a function of wavelength in the close vicinity of $A\;\left(X_{1}\right)$ and $B\;\left(X_{2}\right)$ excitonic emission energies. (F) FDTD‐evaluated DoCP showing the trajectory of the electric field vector in the $E_{x}-E_{y}$ plane and denoting the minor b and major a axes of the resulting ellipses.

To accurately describe our on‐chip proof‐of‐principle device's performance, we implemented FDTD calculations (see Section 4) using the Meep package [52] for multilayer structure of $Si/SiO_{2}/ReSe_{2}/\alpha -MoO_{3}/air$, accounting for the measured critical parameters and multiple reflections in the system, which allowed achieving high consistency between the measured and simulated DoCP signals (Figure 3F, Supporting Information S1: Figure S3F). Here, the emission wavelengths (see Figure 3E), the thicknesses of $ReSe_{2}$ and $\alpha -MoO_{3}$ layers (see Figure 2C,D), the twist angle between the linear polarization direction of $A\;\left(X_{1}\right)$ excitonic emission, and the slow axis of $\alpha -MoO_{3}$ (see Figure 3A), along with its in‐plane anisotropic optical constants (see Figure 1C), were experimentally defined and fitted, leaving only the optical constants of emissive $ReSe_{2}$ approximated. The latter was applied due to a significant spread in values reported in the literature [35, 53, 54], accompanied by a recent demonstration of Hermitian and non‐Hermitian parts of its dielectric permittivity tensor [35, 55], which complicates the creation of an exact optical model for this material. Therefore, we used its approximated optical constants, neglecting the non‐Hermitian part of the permittivity tensor, and included excitons in the modeling as an external source of emission.

Supporting Information S1: Figure S4 presents DoCP and phase retardance maps as a function of the twist angle between $A\;\left(X_{1}\right)$ excitonic emission's linear polarization direction and the slow axis of the polarization‐conversion layer and its thickness. Using measured critical parameters for Device 1 (or Device 2), our model yields elliptical polarization of 54% (or 38%) and 33% (or 18%) for $A\;\left(X_{1}\right)$ and $B\;\left(X_{2}\right)$ state NIR spectral region emissions, which are in good agreement with experimental data considering approximated optical constants of $ReSe_{2}$. The latter were computed accounting for multiple reflections within the multilayer structure resulting in an elliptical shape for $E_{x}-E_{y}$ plane trajectory of electric field's vector with a and b minor and major axes, where DoCP = $2\left(b/a\right)/\left(1+{\left(b/a\right)}^{2}\right)=\left(I_{\sigma_{+}}-I_{\sigma_{-}}\right)/\left(I_{\sigma_{+}}+I_{\sigma_{-}}\right)$ was defined from ellipticity, that is, the ratio between its main axes (see Figure 3F), consistent with the experimental approach. Our evaluations demonstrate that one may further boost the DoCP signal by finer tuning of the twist angle between $ReSe_{2}$’s $A\left(X_{1}\right)$ excitonic state's linear polarization and the slow axis of $\alpha -MoO_{3}$ or an unambiguous thickening of the latter. Notably, in the case of aforementioned parametrization, at $45^{o}$ twist angle (or $30^{o}$ twist angle for Device 2) further increasing $\alpha -MoO_{3}$ layer thickness to 1.45 μm (or 1.35 μm), one may achieve a DoCP close to unity (more than 99 %), yet with twice as thinner thicknesses than free‐standing $\alpha -MoO_{3}$ quarter‐wave plate slabs (see Supporting Information S1: Figures S1 and S4).

## 3 Conclusion

In summary, we presented a direct route toward the creation of on‐chip polarization optical elements based on uniquely twisted all‐vdW heterostructures composed of an emissive $ReSe_{2}$ layer, which served as a source of linearly polarized excitonic emission in the NIR spectral region, and a multilayer $\alpha -MoO_{3}$ slab, which was used as a polarization‐conversion layer. Here, DoCP signals were probed by polarization‐resolved PL mapping, which presented a clear contrast between a bare $ReSe_{2}$ and twisted $ReSe_{2}$/$\alpha -MoO_{3}$ stack's excitonic responses. A consistent FDTD‐based optical model was developed to describe the resulting elliptical polarization and validated using the optical constants of $\alpha -MoO_{3}$ determined by spectroscopic micro‐ellipsometry. Our analysis further indicated that for $\alpha -MoO_{3}$ polarization‐conversion layer incorporated into a twisted vdW stack, achieving a nearly ideal circular polarization state with given device parametrization requires either operation at shorter wavelengths, where the in‐plane optical anisotropy of $\alpha -MoO_{3}$ is larger, or a layer thickness of at least half of that required to satisfy the zero‐order quarter‐wave plate condition in a free‐standing slab. We believe that our findings will be of great use for the understanding of peculiarities in the design of on‐chip polarization optical elements based on all‐vdW twisted heterostructures and can be potentially extended not only to other orthorhombic and triclinic vdW crystals but also applied for a conversion of linearly polarized single photonic emission arising at the surface or in the bulk of some vdW crystals.

## 4 Methods

Sample fabrication: $ReSe_{2}$ crystals were grown by CVT in a quartz ampule using iodine as a transport medium. Rhenium (99.99%, −100mesh, Molymet, Chille) and selenium (99.999%, granules, Retorte, Germany) were placed in a quartz ampule (40 × 250 mm) together with 0.6 g of iodine (99.9%, granules, Fisher Scientific, UK) and melt‐sealed under high vacuum. The ampules were first gradually heated in a muffle furnace at 500°C for 25 hours, 600°C for 50 hours, and 800°C for another 50 hours. The reacted ampules were placed into a two‐zone furnace for crystal growth. The growth zone was first heated up to 1000°C, and the source zone was heated up to 800°C. Subsequently, after 2 days, the thermal gradient was reversed; the source zone was heated up to 1000°C and the growth zone was heated up to 900°C for 14 days. The ampules were cooled to room temperature and opened in an argon‐filled glovebox. $\alpha -MoO_{3}$ crystals were purchased from 2D Semiconductors. Both crystals were micro‐mechanically cleaved into multilayer samples on top of standard $Si/SiO_{2}$ substrates at room temperature using commercial Scotch tape from Nitto Denko Corporation. Prior to micro‐mechanical cleavage, the substrates were sequentially cleaned in acetone, isopropyl alcohol, and deionized water, followed by an air plasma treatment to remove residual surface contaminants and improve the adhesion. The twisted $ReSe_{2}$/$\alpha -MoO_{3}$ vdW heterostructures were prepared using a commercial PDMS stamp‐based deterministic dry‐transfer technique, where the individual layers were aligned with respect to each other by micro‐mechanical control of the twist angle during the stacking.

Spectroscopic micro‐ellipsometry: To accurately access the dielectric tensor components, biaxial $\alpha -MoO_{3}$ samples were studied on 295 nm $Si/SiO_{2}$ and bare Si substrates with their thicknesses preliminarily determined by AFM scanning. The anisotropic optical response was then measured at room temperature using MM imaging spectroscopic micro‐ellipsometry with a Park Systems Accurion EP4 imaging ellipsometer over the 400‐ to 900‐nm spectral region at different angles of incidence (AoI). To further support the subsequent fitting procedure and reduce parameter correlations, the experimental dataset was built from $\alpha -MoO_{3}$ samples of different thicknesses and arbitrary in‐plane orientations on both substrates. Additional MM measurements were collected at 633 nm and an AoI of $55^{o}$ across 20 azimuthal orientations spanning 0‐$180^{o}$. The measured data were then analyzed in EP4Model software using an anisotropic multilayer optical model, in which $\alpha -MoO_{3}$ was treated as a biaxial material with two in‐plane and one out‐of‐plane dielectric tensor components. Given the negligible Vis spectral region absorption, its optical response was treated as lossless and both the in‐plane and out‐of‐plane components were described using Sellmeier's dispersion relation: $n_{i}^{2}$(λ) = 1 + $B_{i}$ $\lambda^{2}$/($\lambda^{2}$‐$C_{i}$), where i = x, y, z (Sellmeier's coefficients obtained from the fit are summarized in Supporting Information S1: Table S1).

Polarization‐resolved Raman spectroscopy: Raman spectra from $\alpha -MoO_{3}$ samples were acquired using a custom‐designed system comprising a confocal microscope equipped with a 100 X objective lens (N.A. = 0.70) and a PyLoN 100‐BRX LN2‐cooled CCD camera coupled to a SpectraPro HRS‐750 spectrometer. The measurements were carried out using a 532‐nm CW laser and a RazorEdge LP03‐532RE‐25 long‐pass filter. The polarization angle was varied from $0^{o}$ to $360^{o}$ with a step of $5^{o}$ by simultaneous rotation of half‐wave plates in the excitation and detection paths, while in the detection path, the position of the polarizer was fixed.

Micro‐reflection spectroscopy: Vis micro‐reflection spectroscopy of $\alpha -MoO_{3}$ samples was performed by utilizing a commercial optical microscope (Nikon Eclipse LV100ND) equipped with polarizers in the excitation and detection paths aligned at $90^{o}$ and a green bandpass filter in the excitation path. To control the rotation angle of the investigated samples, a rotational mount from Thorlabs components was used.

Polarization‐resolved PL spectroscopy: To perform polarization‐resolved PL measurements, the samples were loaded into a dry cryogenic system with a base temperature of 4 K and mounted onto x‐y‐z piezo‐stages. The measurements were realized in a back‐scattering configuration. The laser focusing and collection were done by a cold objective with N.A. = 0.82. For the detection of the signal, the light was dispersed by a SpectraPro HRS‐750 spectrometer (Princeton Instruments) and detected by a PyLoN 100‐BRX LN2‐cooled CCD camera (Princeton Instruments). Depolarized light was used for the measurements of DoCP signals. For the collection of the signal, a quarter‐wave plate was placed in the sequence with a polarizer in front of the spectrometer. A half‐wave plate was placed in the sequence with a polarizer in front of the spectrometer for the collection of DoLP signals.

Atomic‐force microscopy: Topography measurements were performed using Bruker's Dimension FastScan XR atomic‐force microscope operated in tapping and peak‐force modes with RTESPA‐150 cantilevers.

TMM and FDTD calculations: TMM studies were used to estimate the thickness of $\alpha -MoO_{3}$ used as polarization‐conversion layer for a system containing three layers: air/$\alpha -MoO_{3}$/air, where the optical constants of $\alpha -MoO_{3}$ were taken according to the ellipsometry fit at a wavelength of 884.4nm: $n_{x}=2.26$, $n_{y}=2.18$, and $n_{z}=1.94$. FDTD calculations were performed using fitted experimental parameters of both multilayer $Si/SiO_{2}/ReSe_{2}/\alpha -MoO_{3}/air$ devices, where the optical model consisted of 285nm thick $SiO_{2}$, 6nm (or 70nm) thick $ReSe_{2}$ layer, and 1400nm (or 910nm) thick $\alpha -MoO_{3}$ layer, placed onto Si substrate. The optical constants of the latter were taken in accordance with an ellipsometric fit for wavelengths corresponding to the excitonic states in each $ReSe_{2}$ sample. The twist angle between the linearly polarized excitonic emission state of $A\;\left(X_{1}\right)$ and the slow axis of $\alpha -MoO_{3}$ was taken as 41° (or 21° for Device 2). See Supporting Information S1: Table S2 for the full list of parameters. The space‐grid had 1nm resolution, and the time step was $6.6\cdot 10^{-19}\;s$. The field monitor was placed at a distance of 3μm above the $\alpha -MoO_{3}$ layer, capturing $E_{x}$ and $E_{y}$ components every 0.07fs, which resulted in about 50 points per period at a given frequency. The resulting trajectory of the electric field vector in the $E_{x}-E_{y}$ plane after multiple reflections had the shape of an ellipse, where DoCP was defined from ellipticity, that is, the ratio between its main axes as $2\left(b/a\right)/\left(1+{\left(b/a\right)}^{2}\right)$. Phase retardance δ was recalculated from b/a and an angle between $\alpha -MoO_{3}$ slow axis and polarization direction θ from an equation:

$${\left(\frac{b}{a}\right)}^{2}=\frac{1/\cos{\left(\theta \right)}^{2}+1/\sin{\left(\theta \right)}^{2}-B\left(\theta ,\delta \right)}{1/\cos{\left(\theta \right)}^{2}+1/\sin{\left(\theta \right)}^{2}+B\left(\theta ,\delta \right)},\;\mathrm{where}\;B\left(\theta ,\delta \right)=\sqrt{{\left(1/\cos{\left(\theta \right)}^{2}-1/\sin{\left(\theta \right)}^{2}\right)}^{2}+4{\left(\frac{\cos\left(\delta \right)}{\cos\left(\theta \right)\sin\left(\theta \right)}\right)}^{2}}$$

## Author Contributions

**Dmitrii Litvinov:** writing – original draft, investigation, formal analysis, data curation, visualization, writing – review and editing, methodology, validation. **Ilia Begichev:** writing – original draft, writing – review and editing, formal analysis, visualization, software, validation, data curation. **Christoph Reuter:** investigation, formal analysis, data curation. **Changyi Chen:** formal analysis, investigation, data curation. **Iakov Reznikov:** investigation, data curation. **Maksim Sargsyan:** investigation, formal analysis, data curation, writing – original draft. **Sergey Y. Grebenchuk:** investigation, data curation, formal analysis. **Zdeněk Sofer:** investigation, resources, formal analysis. **Makars Šiškins:** investigation, data curation, formal analysis, validation. **Kostya S. Novoselov:** supervision, funding acquisition, conceptualization, project administration. **Alexey I. Berdyugin:** funding acquisition, supervision, conceptualization, project administration. **Maciej Koperski:** conceptualization, funding acquisition, writing – review and editing, supervision, project administration, writing – original draft. **Davit A. Ghazaryan:** supervision, writing – original draft, conceptualization, funding acquisition, writing – review and editing, project administration.

## Conflicts of Interest

The authors declare no conflicts of interest.

## Supporting information


Supporting Information S1

## Data Availability

All data supporting the findings of this article and its Supporting Information will be made available upon request to the corresponding authors.
