# Supplementary material for: Toward On‐Chip Nascent All‐van‐der‐Waals Polarization Optical Elements for Nanoscale Photonic Applications
Source: Nanophotonics. 2026 Oct 24;15(20):e70327. doi: 10.1002/nap2.70327 (PMC13695083; doi:10.1002/nap2.70327)
Supplement: Supplementary file 1 [file NAP2-15-e70327-s001.pdf]

Supporting Information for: Towards on-chip nascent all-van-der-Waals polarization optical elements for nanoscale photonic applications

Dmitrii Litvinov[1,2,†], Ilia Begichev[1,2,†], Christoph Reuter[1,2], Changyi Chen[1,2], Iakov Reznikov[1,2], Maksim Sargsyan[3], Sergey Y. Grebenchuk[1], Zdeněk Sofer[4], Makars Šiškins[1,5], Kostya S. Novoselov[1,2], Alexey I. Berdyugin[1,2,6], Maciej Koperski[1,2,*] and Davit A. Ghazaryan[1,3,*]

[1]**Institute for Functional Intelligent Materials, National University of Singapore, 117544, Singapore**

[2]**Department of Materials Science and Engineering, National University of Singapore, 117575, Singapore**

[3]**Laboratory of Advanced Functional Materials, Yerevan State University, 0025 Yerevan, Republic of Armenia**

[4]**Department of Inorganic Chemistry, University of Chemistry and Technology Prague, Prague 166 28, Czech Republic**

[5]**School of Physics and Astronomy, University of Southampton, Highfield, SO17 1BJ Southampton, United Kingdom**

[6]**Department of Physics, National University of Singapore, Singapore 117575, Singapore**

1 |

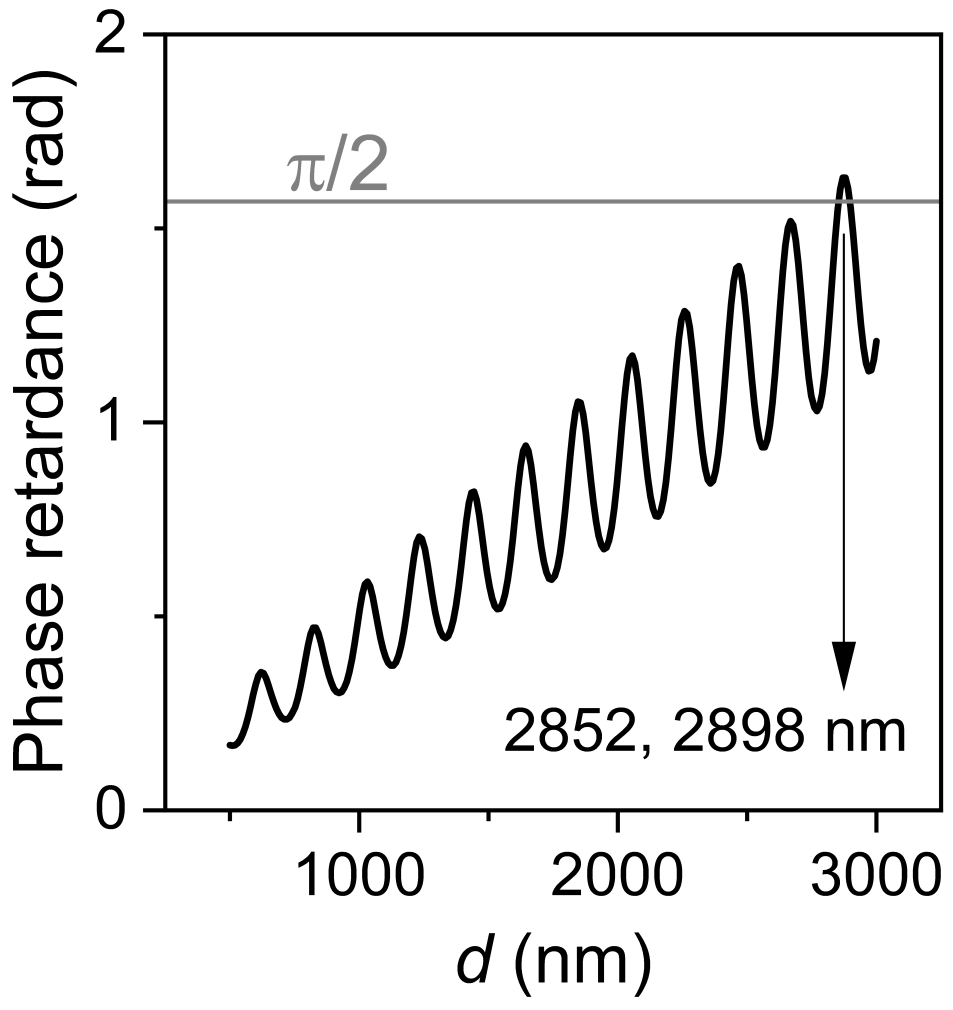

FIGURE 1 TMM calculated phase retardance for air/$\alpha - MoO_3$/air structure at the wavelength of 884.8 nm.

2 |

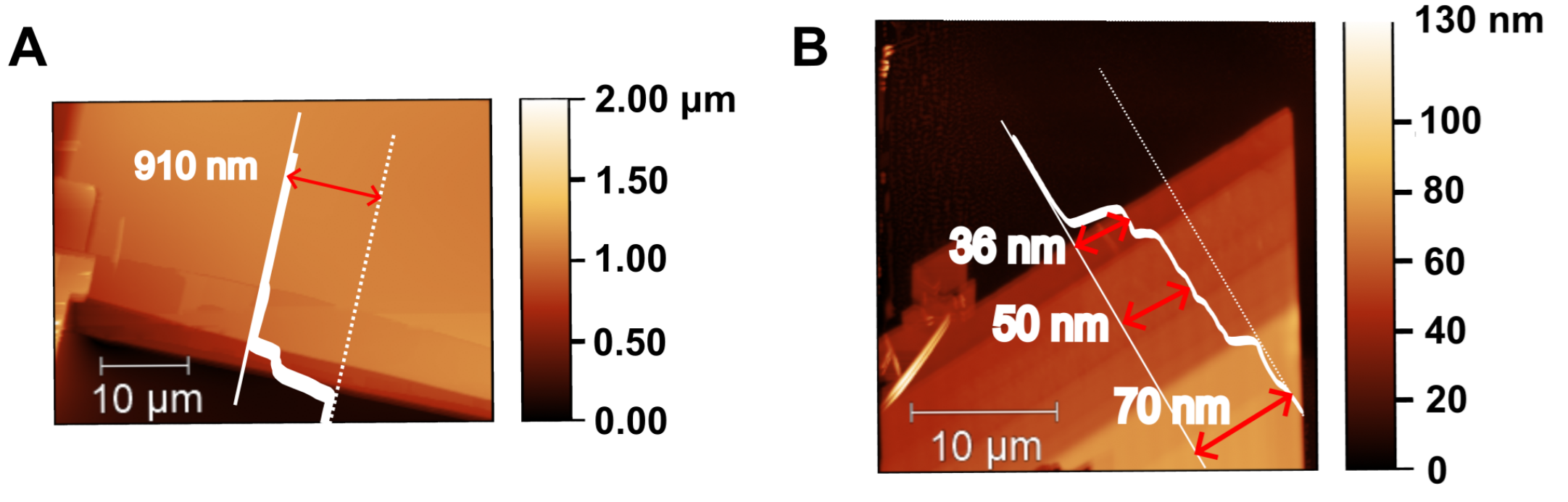

FIGURE 2 Thickness characteristics of biaxial layers selected for the design of the on-chip polarization conversion elements, Device 2. (A) and (B) AFM maps and thickness profiles (shown by red arrows) corresponding to cross-sections of $\alpha - MoO_3$ (C) and $ReSe_2$ (D). 70 nm thick $ReSe_2$'s terrace was used for the characterization of Device 2. All the samples presented throughout the figure were placed onto Si/SiO_2 substrates.

TABLE 1 *The fitted Sellmeier parameters for vdW $\alpha - MoO_3$ along the x, y, and z crystallographic axes.*

Component	Axis i	B_i	C_i $10^{-2}(\mu m^2)$
Out-of-plane	z	2.639	4.0
In-plane	y	3.563	3.63
In-plane	x	3.834	4.923

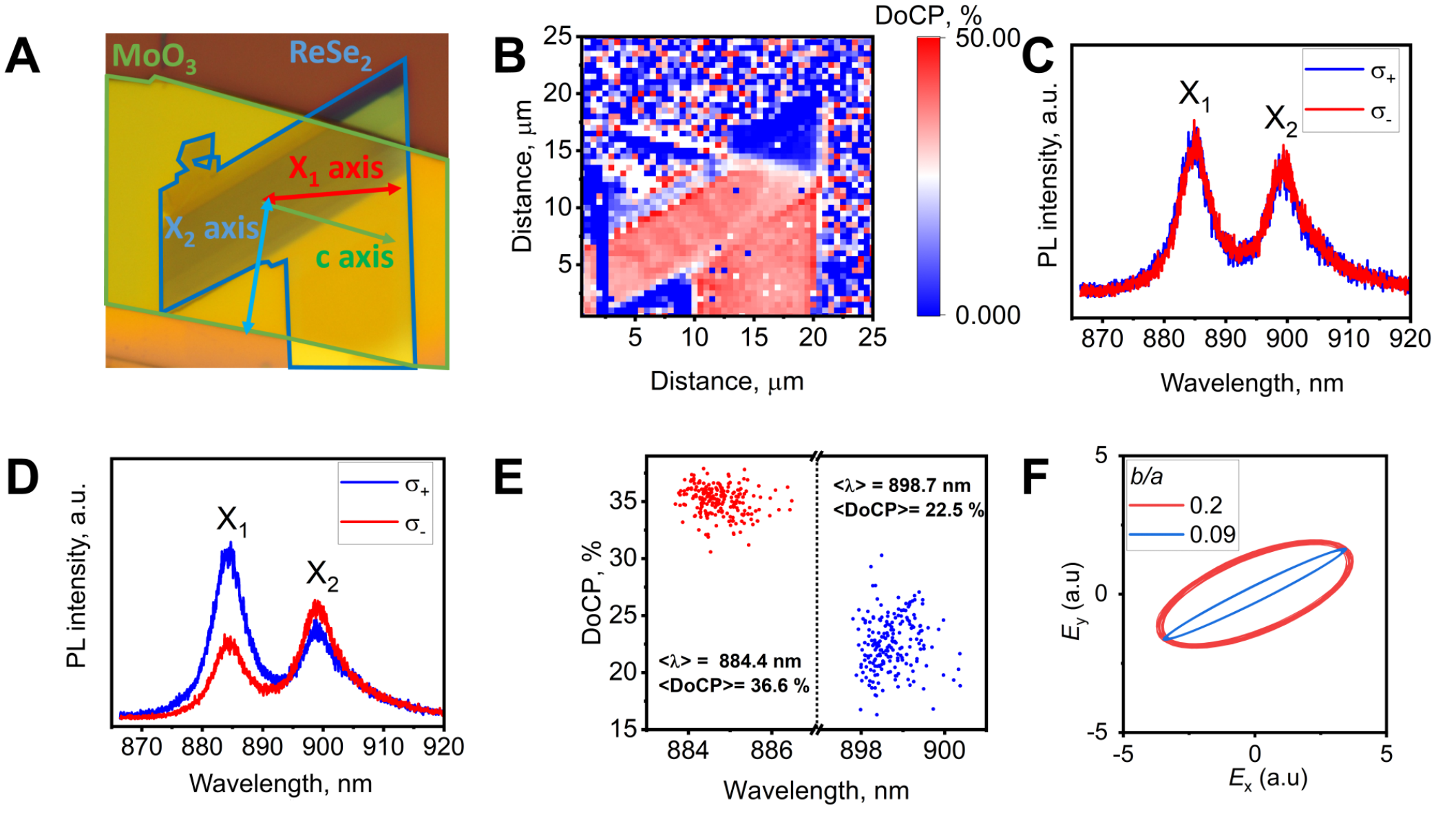

FIGURE 3 Probing DoCP in $ReSe_2/\alpha - MoO_3$ twisted heterostructure assembled on top of Si/SiO_2 chip, Device 2. (A) 50 X optical micrograph of proof-of-principle sample. Blue arrow denotes the linear polarization direction of $A\,(X_1)$ excitonic emission from $ReSe_2$, while green arrow shows the slow axis of $\alpha - MoO_3$ layer. (B) DoCP map collected all over the sample, including bare $ReSe_2$ and substrate. (C) and (D) An example of circularly polarized σ_+ and σ_- state PL spectra acquired from bare $ReSe_2$ sample of 70 nm in thickness (C) and $ReSe_2/\alpha - MoO_3$ twisted heterostructure (D). Excitonic states are denoted as X_1 and X_2. (E) DoCP histograms collected from twisted stack as a function of wavelength (in a close vicinity to $A\,(X_1)$ and $B\,(X_2)$ excitonic emission energies). (F) FDTD evaluated DoCP showing the trajectory of electric field's vector in $E_x - E_y$ plane, denoting the minor a and major b axes of resulting ellipses.

TABLE 2 *Parameters of optical model used in FDTD and comparison of DoCP obtained from theory and from experiment.*

	D1 exciton A	D1 exciton B	D2 exciton A	D2 exciton B
Wavelength (μm)	0.880	0.896	0.884	0.900
θ	41	62	21	81
DoCP from experiment	64.5	37	36.5	22.5
DoCP from FDTD	54	33	38	18

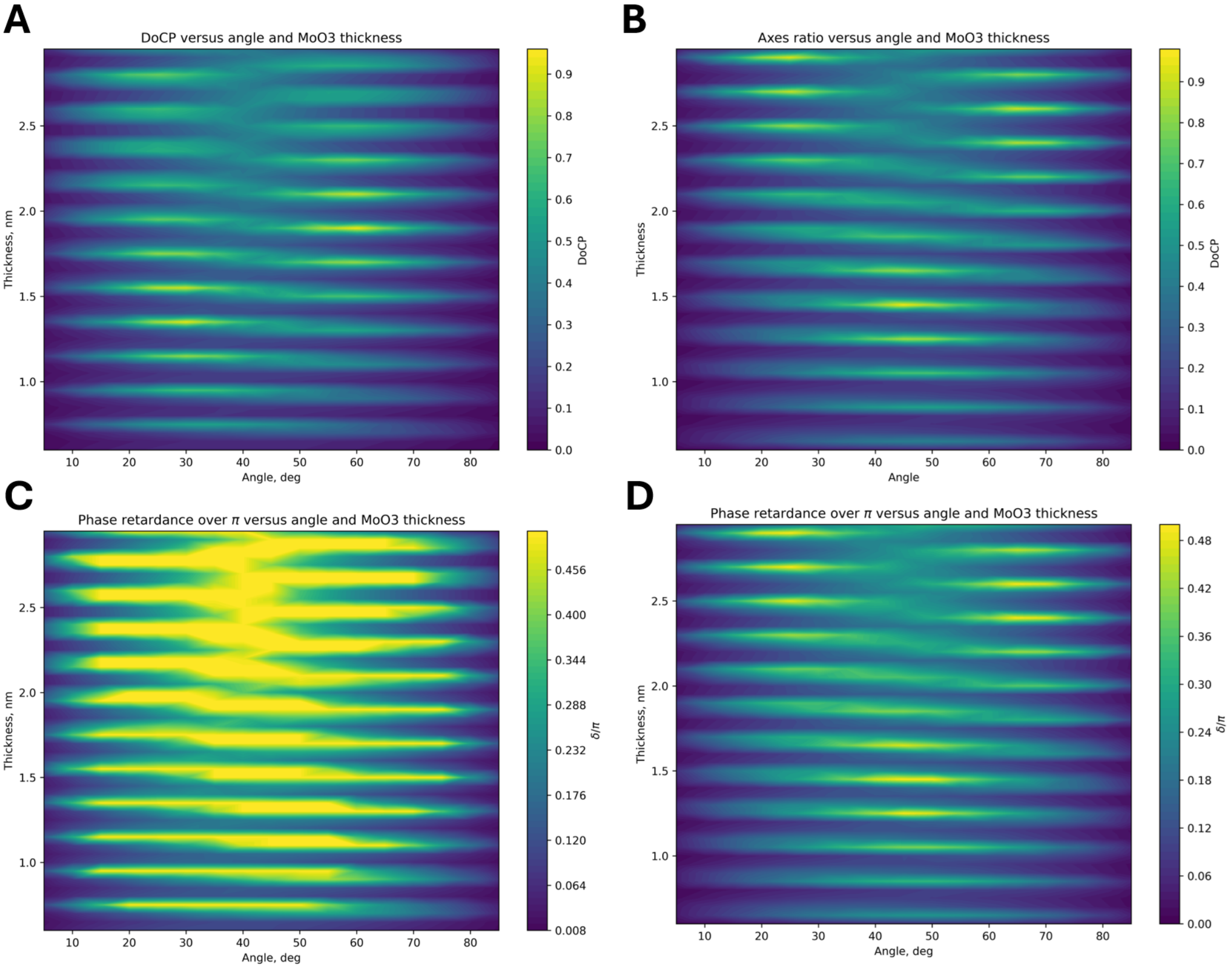

FIGURE 4 FDTD calculated (A) and (B) DoCP and normalized (over π) phase retardance (C) and (D) contour plots as function of $\alpha - MoO_3$ polarization-conversion layer's thickness and the twist-angle between linearly polarized A excitonic emission of $ReSe_2$ and the slow axis of the former. (A) and (C) show the characteristics of Device (70 nm/910 nm $ReSe_2/\alpha - MoO_3$), (B) and (D) present the characteristics of Device 1 (6 nm/1400 nm $ReSe_2/\alpha - MoO_3$) for wavelengths corresponding to A excitonic state energies. DoCP maximal values achieved for twist angles shifted from a classical 45° in Device 2 (and Device 1 in the case of thicknesses more than half of zero-order quarter-wave plate conditions of a free-standing slab) are attributed to the presence of two twisted biaxial layers in all-vdW stacks.
